# Increased plasma levels of glucose-dependent insulinotropic polypeptide are associated with decreased postprandial energy expenditure after modern Japanese meals

**DOI:** 10.1007/s00394-016-1216-y

**Published:** 2016-04-25

**Authors:** Noriko Osaki, Chika Suzukamo, Kouji Onizawa, Tadashi Hase, Akira Shimotoyodome

**Affiliations:** Biological Science Laboratories, Kao Corporation, 2606 Akabane, Ichikai-machi, Haga-Gun, Tochigi 321-3497 Japan

**Keywords:** Gastrointestinal hormones, Ghrelin, Glucose-dependent insulinotropic polypeptide, Japanese meal, Postprandial energy expenditure

## Abstract

**Purpose:**

The nutritional changes that have accompanied the modernization of Japanese dietary patterns have led to significant increases in the number of people who are overweight or obese. This study aimed to clarify the effects of these nutritional changes on postprandial energy expenditure and the release of metabolism-regulating hormones.

**Methods:**

The total daily energy content (20 % breakfast, 40 % lunch, and 40 % dinner) and macronutrient composition (carbohydrate/fat/protein) was 8807.3 kJ and 364.3:30.1:66.4 (g) for the traditional test diet and 9217.6 kJ and 331.7:66.1:76.9 (g) for the modern test diet. In experiment 1, nine healthy Japanese men participated in a crossover study during which they ingested a test diet comprising three meals; postprandial blood parameters were measured after each meal. In experiment 2, another ten men participated in a crossover study during which they ingested 2 meals, after which metabolic responses and blood variables were evaluated.

**Results:**

The modern diet induced greater blood levels of glucose-dependent insulinotropic polypeptide (GIP) and ghrelin than did the traditional diet. The expected increase in postprandial energy expenditure (∆REE) tended to be dampened after the modern compared with the traditional diet. GIP was inversely correlated with ∆REE after lunch, and ghrelin was positively associated with ∆REE.

**Conclusion:**

Both GIP and ghrelin are robust indicators of postprandial energy expenditure. The nutritional changes accompanying the modernization of Japanese dietary patterns may increase the levels of the anabolic intestinal hormone GIP, which is associated with ∆REE, in the Japanese population. The contribution of an increased ghrelin concentration to the decreased ∆REE after the modern diet warrants further investigation.

## Introduction

Obesity increases the risk of several health conditions, including hypertension, hyperlipidemia, and type 2 diabetes [1], and significantly increases healthcare costs. The World Health Organization (WHO) reported that more than one billion adults were overweight worldwide in 2009, with more than 300 million of them being clinically obese. According to WHO criteria, Japan had the lowest rate of obesity (approximately 3.9 %) among the OECD member countries in 2009 [2]. The onset and progression of obesity are closely related to lifestyle habits involving dietary patterns, physical activity, rest, tobacco use, and alcohol consumption.

Traditional Japanese dietary patterns are characterized by the high consumption of soybean products, fish, root crops, seaweeds, mushrooms, vegetables, fruits, and green tea [3]. Many epidemiologic studies have shown that the consumption of these food items or the adherence to a traditional Japanese dietary pattern in itself is inversely associated with lifestyle-related illnesses, such as cardiovascular disease, cancer, high blood pressure, high serum lipid levels, and diabetes [4–7]. In addition, the traditional Japanese diet has drawn considerable attention since the 1960s because of its association with an extremely low rate of coronary heart disease [8].

However, the National Health and Nutrition Survey has revealed that dietary patterns in Japan are becoming increasingly westernized. The consumption of rice (a conventional staple food for Japanese), soy, and fish has been decreasing since the 1960s, such that meat and products containing wheat flour are now preferred by the Japanese population. Although the average total energy intake has not changed significantly, the consumption of dietary fat, especially animal fat, by the Japanese population has increased [3, 9]. Along with the observed changes in dietary nutrient composition, the number of overweight and obese Japanese is increasing, especially among men in their 30s to 60s. The 2008 report of the National Health and Nutrition Survey stated that 28.6 % of men and 20.6 % of women in Japan were considered to be obese or overweight [10]. Whereas an adverse dietary nutrient composition such as high fat content is known to increase the risk of obesity, how changes in nutrient composition affect energy balance in humans has yet to be elucidated.

Several mechanisms may explain the effect of nutrient composition on energy balance. One potential mechanism may involve the release of gastrointestinal hormones by specialized enteroendocrine cells after the ingestion of nutrients. Recent studies have shown that gastrointestinal hormones play an important role in regulating energy balance. For example, peptide YY (PYY) [11, 12], ghrelin [13–16], glucose-dependent insulinotropic polypeptide (GIP) [17–27], and glucagon-like peptide 1 (GLP-1) [17, 21, 25–27] are known to regulate energy metabolism and appetite. Accordingly, these hormones have been the focus of increased interest due to their therapeutic potential for the treatment of obesity. In addition, several studies have proposed that dietary nutrient composition may influence the postprandial energy balance by regulating gastrointestinal hormones.

Therefore, the principal aim of the current study was to clarify how the nutritional changes associated with the modernization of the Japanese diet affect postprandial energy metabolism and gastrointestinal hormone responses. To this end, we compared postprandial energy expenditure and the postprandial blood levels of various metabolism-regulating hormones in healthy Japanese men after their consumption of traditional or modern Japanese meals.

## Materials and methods

### Subjects

Healthy Japanese adult men (age, 20–59 years) were recruited for this study. Subjects provided written informed consent prior to the study. Subjects had no history of diabetes mellitus or food allergies and showed no abnormalities on physical examination.

This study was conducted according to the guidelines of the Declaration of Helsinki, and all procedures involving human subjects were approved by the Ethics Committee of Kao Corporation.

### Experimental design

All subjects abstained from alcohol and exercise for 2 days before beginning the study. The evening before each test day, subjects consumed the same dinner (fat content, 25.4 g; total energy content, 3740 kJ [894 kcal]) before 21:00 h; participants abstained from additional food intake for at least 12 h and from ingesting water for at least 3 h before testing. Subjects arrived at the test site between 08:00 and 08:30 h of the test day.

### Test meals

Test meals were prepared to reflect the average nutrient composition of the Japanese diet according to the National Health and Nutrition Survey in Japan of 1960 (traditional test diet) and 2008 (modern test diet). For the traditional test diet, breakfast consisted of polished rice, miso soup, natto, and Japanese pickles. Lunch comprised polished rice, miso soup, mackerel, boiled vegetables, bean curd (tofu), an orange, and Japanese pickles. Dinner consisted of polished rice, miso soup, sardines, boiled vegetables (squash and broccoli), and boiled kidney beans with dressing. The total energy content of the 3-meal traditional test diet was 8807.3 kJ (20 % breakfast, 40 % lunch, and 40 % dinner), and the macronutrient composition (carbohydrate/fat/protein) was 364.3:30.1:66.4 g (energy %, 73.1:13.6:13.3). The total energy content of the 2-meal traditional test diet was 5333.2 kJ (31 % breakfast and 69 % lunch), and the macronutrient composition (carbohydrate/fat/protein) was 216.8:19.7:39.2 g (energy %, 72.2:14.8:13.1).

For the modern Japanese test diet, breakfast consisted of toast, ham salad, apple juice, and fruit yogurt. Lunch comprised pork curry and rice, tuna salad, yogurt, and pieces of pineapple. Dinner consisted of polished rice, miso soup, beef and pork patties, spinach, squash with cheese, and an orange. The total energy content of the 3-meal modern Japanese test diet was 9217.6 kJ (20 % breakfast, 40 % lunch, and 40 % dinner), and its macronutrient composition (carbohydrate/fat/protein) was 331.7:66.1:76.9 g (energy %, 59.5:26.7:13.8). The total energy content of the 2-meal modern test diet was 5911.2 kJ (34 % breakfast and 66 % lunch), and the macronutrient composition (carbohydrate/fat/protein) was 195.3:41.1:48.3 g (energy %, 58.1:27.5:14.4). The energy content and macronutrient composition of the test meals were calculated according to the Standard Tables of Food Composition in Japan 2010.

### Experiment 1: Evaluation of blood parameters after consumption of traditional compared with modern Japanese test meals

Nine male volunteers (age, 32.6 ± 2.3 year; body weight, 72.8 ± 4.6 kg; body mass index, 23.7 ± 1.4 kg/m^2^) participated in the study.

At 08:15 h, before the ingestion of the first test meal, blood samples (approximately 200 μL) for analysis of fasting blood parameters were collected into heparinized capillary tubes (75 mm length; Drummond Scientific, Broomall, PA, USA) by pricking a finger with a single-use sterile capillary blood sampling device (BD Genie Lancet, Becton–Dickinson, Tokyo, Japan). Blood samples were transferred into capillary blood collection tubes (CapiJect with EDTA-2Na, Terumo Medical, Tokyo, Japan) containing dipeptidyl peptidase IV inhibitor (Millipore, Tokyo, Japan) and maintained on ice until plasma preparation. All subsequent blood samples were obtained in the same way and volume, thus yielding at least 90 μL plasma per sample, which was the minimum needed to measure all blood variables in duplicate by using the multiplex hormone assay. Breakfast was served at 08:30 h, lunch at 12:30 h, and dinner at 18:30 h. Subjects ingested each test meal within 10–15 min. The test diets were given in a random order (traditional or modern), with an interval of at least 7 days between diets. Over the postprandial period, blood samples were taken at 09:00, 09:30, and 10:30 h (after breakfast); 12:30, 13:00, 13:30, 14:30, and 16:30 h (at or after lunch); and 18:30, 19:00, 19:30, 20:30, and 21:30 h (at or after dinner). After centrifugation, plasma was stored at −80 °C until analysis.

### Experiment 2: Assessment of energy metabolism after ingestion of traditional compared with modern Japanese test meals

Ten male volunteers (age 36.8 ± 2.4 year; body weight 75.8 ± 4.4 kg; body mass index 24.9 ± 2.1 kg/m^2^) participated in the study.

At 08:30 h (before breakfast), baseline blood samples (approximately 3 mL) were collected from a central vein into tubes containing proprietary stabilizers (P700 tubes, Becton–Dickinson). The blood samples were supplemented with a protease inhibitor (P2714-BTL, Sigma, Tokyo, Japan) and serine protease inhibitor (Pefabloc SC, Roche Diagnostics, Tokyo, Japan) and were maintained at room temperature until plasma preparation. Breakfast was served at 09:00 h and lunch at 13:15 h. Subjects ingested each test meal (prepared as described for experiment 1) within 10–15 min. The test diets were given randomly and unblinded (traditional or modern), with an interval of at least 7 days between diets. During the postprandial period, blood samples were collected at 10:00 h (after breakfast) and at 13:00, 14:15, 17:15, and 19:15 h (after lunch). After centrifugation, plasma was stored at −80 °C until analysis.

During the experimental period, indirect calorimetry was performed 8 times: before breakfast (−15 to 0 min); at 30–45, 60–75, and 240–255 min after breakfast; and at 30–45, 60–75, 240–255, and 360–375 min after lunch. Briefly, respiratory metabolic performance in the sedentary condition was measured by using an open-circuit ventilated-hood system (VO2000, Medical Graphics, St. Paul, MN, USA). Gas exchange rates were recorded on every third expiration. During each 15-min session, baseline data were collected during the first 5 min (equilibration period); data collected during the remaining 10 min were averaged to calculate resting energy expenditure (REE, kJ/day) and the respiratory exchange ratio (RER). The measured values of oxygen consumption (VO_2_) and carbon dioxide production (VCO_2_) were converted to REE according to the Weir equation [28] by using a specialized software program (m-Graph, S&ME, Tokyo, Japan). The software also was used to estimate the respiratory exchange ratio (that is, VCO_2_/VO_2_) and substrate utilization from the gas-exchange data.

The physical activity of the subjects during the experimental period was monitored by using an Actimarker (model EW48001, Panasonic Electric Works, Osaka, Japan) attached to the right hip. The device had a built-in triaxial accelerometer, which measured the 3 axes of acceleration (xi, up and down; yi, left and right; zi, forward and backward).

### Blood analysis

Blood glucose was determined by using a blood glucose self-monitoring device (Accu-Check Comfort, Roche Diagnostics) immediately after blood collection. Plasma levels of TG and non-esterified fatty acids (NEFA) were determined by using spectrophotometric methods (triglyceride E-test and NEFA-C test assay kits, Wako Pure Chemical Industries, Osaka, Japan). Plasma insulin, active ghrelin, PYY, and pancreatic peptide (PP) were measured on an automated system (Luminex 100 Total System, Luminex, Austin, Texas, USA) using Bio-Plex 200 reagents (Bio-Rad, Hercules, CA, USA) and the Milliplex Map Kit Human Gut Hormone Panel (Millipore). Total GIP and active GLP-1 were measured by using an ELISA Kit (Linco Research, St. Charles, MO, USA). Plasma cholecystokinin (CCK) was determined by using an enzyme immunoassay kit (Cholecystokinin Octapeptide; 26–33; non-sulfated; human, rat, mouse; Phoenix Pharmaceuticals, Burlingame, CA, USA).

### Statistical analysis

Numerical data are expressed as mean ± SD. All postprandial blood analyte concentrations were compared with baseline data by using repeated-measures ANOVA in Graph Pad Prism 6 (MDF, Tokyo, Japan) to evaluate the effects of time and each type of meal when the data were normally distributed. Post-breakfast changes were calculated by using the values at 0 h (immediately before breakfast consumption) as baseline, post-lunch changes were determined relative to values at 4 h (immediately before lunch consumption), and post-dinner changes used the values at 10 h (immediately before dinner consumption) as baseline. In addition, incremental areas under the curve (iAUCs) were calculated for blood parameters and ∆REE by using the trapezoidal method [29], whether cumulatively or separately for each the post-breakfast, post-lunch, and the post-dinner. Wilcoxon signed-rank tests and Spearman’s rank correlation coefficients were used when comparing paired samples whose data were not normally distributed (EXCEL Multiple Classification Analysis version 5.0, Esumi, Tokyo, Japan). Differences were considered significant when the error probability was <0.05.

## Results

### Differences in postprandial blood responses between traditional and modern Japanese test meals

**Fig. 1:**
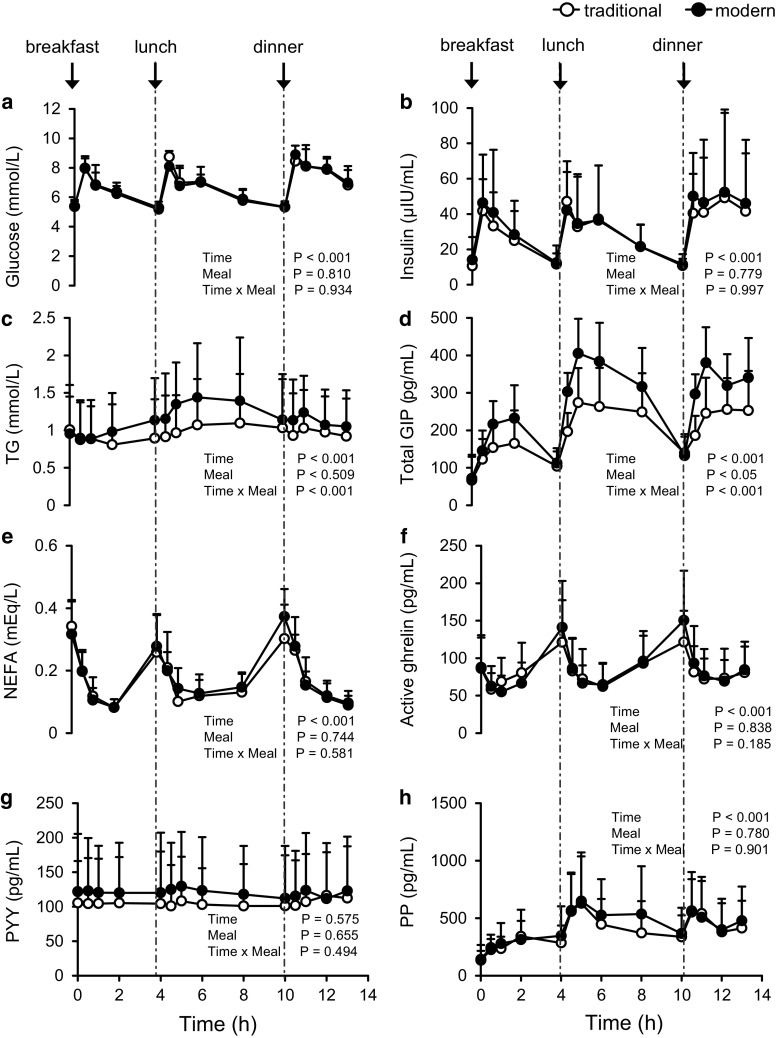
Blood glucose (**a**), insulin (**b**), triglycerides (TG; **c**), total glucose-dependent insulinotropic polypeptide (GIP; **d**), non-esterified fatty acids (NEFA; **e**), active ghrelin (**f**), peptide YY (PYY; **g**), and pancreatic peptide (PP; **h**) concentrations until 13 h after the consumption of the traditional Japanese test diet (*open circles*) or modern Japanese test diet (*closed circles*) in overnight-fasted healthy Japanese men. Data are expressed as mean ± SD (*n* = 9 in each group) and were analyzed by means of 2-way repeated-measures ANOVA

Baseline data from the nine Japanese adult male participants did not differ between the test diets (Fig. 1). Within each diet, all test parameters except PYY (Fig. 1g) demonstrated significant (*P* < 0.001) differences between baseline and postprandial concentrations.

In particular, total GIP concentrations throughout the study differed significantly (*P* < 0.05) between the traditional and modern Japanese test diets (Fig. 1d). Postprandial TG (Fig. 1c) and total GIP (Fig. 1d) levels demonstrated significant (*P* < 0.001) interaction between time and diet. Plasma total GIP concentrations increased after each meal and peaked within 2 h after breakfast and 1 h after lunch and dinner and did not decline to basal (i.e., pre-meal) levels. The iAUCs of total GIP after each meal (breakfast, lunch, and dinner) were higher after the modern diet than after the traditional diet, whereas those of blood TG did not differ significantly between the test diets (Table 1). The postprandial total GIP responses (iAUCs) after the breakfast, lunch, and dinner of the modern Japanese test diet were 153, 145, and 241 % of those after the traditional Japanese meals (Table 1).

**Table 1:** Postprandial incremental areas under the curve (iAUCs) of blood glucose, TG, insulin, and total GIP

		Traditional	Modern
*Glucose*			
iAUC (after breakfast)	(mmol 4 h/L)	3.90 (1.30)	3.73 (1.58)
iAUC (after lunch)	(mmol 6 h/L)	5.47 (2.36)	5.48 (2.46)
iAUC (after dinner)	(mmol 3 h/L)	6.75 (0.78)	7.33 (1.58)
*TG*			
iAUC (after breakfast)	(mmol 4 h/L)	0.08 (0.15)	0.26 (0.32)
iAUC (after lunch)	(mmol 6 h/L)	0.91 (0.64)	1.27 (1.26)
iAUC (after dinner)	(mmol 3 h/L)	0.12 (0.13)	0.14 (0.15)
*Insulin*			
iAUC (after breakfast)	(μIU 4 h/mL)	55.39 (39.09)	56.50 (44.17)
iAUC (after lunch)	(μIU 6 h/mL)	91.21 (73.95)	86.17 (70.63)
iAUC (after dinner)	(μIU 3 h/mL)	91.10 (95.36)	103.72 (85.75)
*Total GIP*			
iAUC (after breakfast)	(pg 4 h/mL)	283.36 (122.33)	433.44 (206.73)*
iAUC (after lunch)	(pg 6 h/mL)	890.93 (254.31)	1287.68 (662.11)†
iAUC (after dinner)	(pg 3 h/mL)	274.28 (151.01)	662.11 (149.84)†

Data are expressed as means (SD); *n* = 9 in each group and were analyzed by using the Wilcoxon signed-rank test

*GIP* glucose-dependent insulinotropic polypeptide, *TG* triglycerides

Values significantly (* *P* < 0.05, † *P* < 0.01) different from those of the traditional Japanese test diet are indicated

Unlike TG and GIP concentrations, blood levels of glucose (Fig. 1a), insulin (Fig. 1b), NEFA (Fig. 1e), active ghrelin (Fig. 1f), PYY (Fig. 1g), and PP (Fig. 1h) throughout the study did not differ significantly between the test diets. Furthermore, the iAUCs for blood glucose, TG, and insulin after each meal (breakfast, lunch, and dinner) did not differ significantly between the test diets (Table 1), nor did those for NEFA, active ghrelin, PYY, and PP (data not shown).

### Differences in postprandial energy expenditure between traditional and modern Japanese diets

All participants completed all trials (*n* = 10); however, the measurement was not stable for the bad condition of VO2000 in one participant, and data for indirect calorimetry evaluation and blood responses are presented from 9 participants. The initial energy metabolism and blood variables were similar between the test diets.

The physical activity of subjects did not differ between the test diets (data not shown).

**Fig. 2:**
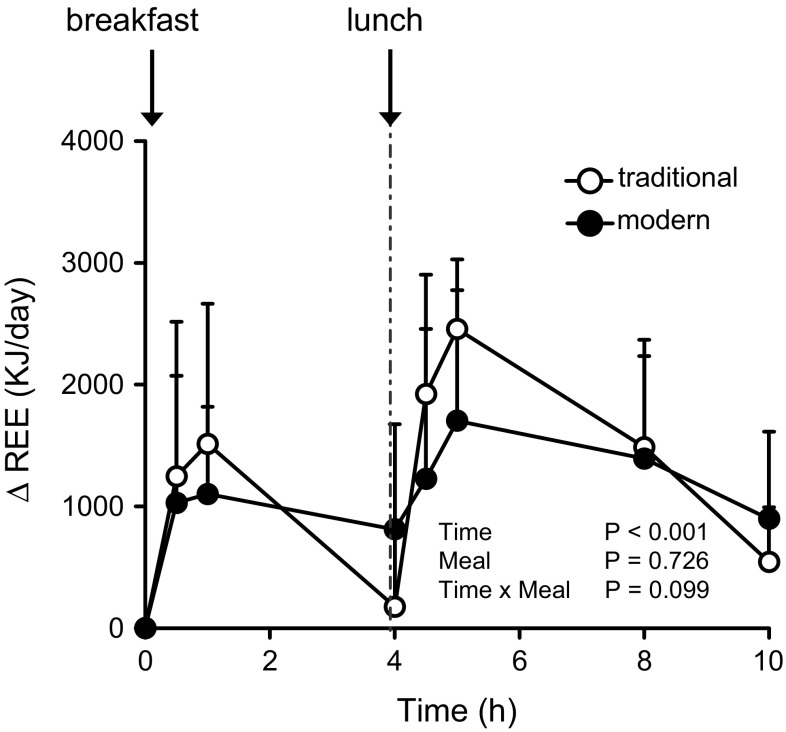
Postprandial incremental resting energy expenditure (∆REE) after the consumption of the traditional Japanese test diet (*open circles*) or modern Japanese test diet (*closed circles*) in overnight-fasted healthy Japanese men. Baseline values were obtained immediately before the ingestion of the breakfast meal. Data are expressed as mean ± SD (*n* = 9 in each group) and were analyzed by means of 2-way repeated-measures ANOVA

Compared with that of the traditional diet, the postprandial increase in REE (∆REE) of the modern diet tended to be lower soon after the meal ingestion but higher during the later hours, but the difference between the diets at each postprandial time point was not significant (*P* > 0.05) (Fig. 2). The incremental area under the curve (iAUC) of ∆REE at 4 h after breakfast did not differ significantly between the test diets. In contrast, the iAUC of ∆REE at 6 h after lunch was significantly (*P* < 0.05) lower after the modern Japanese test meals than after the traditional ones when the values immediately before lunch (at 4 h) were used as the baseline levels (Table 2). However, when the values immediately before breakfast (at 0 h) were used as the baseline levels, the iAUC of ∆REE after lunch did not differ between the test diets (Table 2).

**Table 2:** Postprandial incremental REE (iAUCs) after the test diets

	Traditional	Modern	*P*
After breakfast (0–4 h) (kJ)	574.6 (513.7)	619.5 (454.7)	0.95
After lunch (4–10 h) (kJ): the baseline levels were used at 4 h	2211.7 (632.7)	791.4 (637.3)	<0.05
After lunch (4–10 h) (kJ): the baseline levels were used at 0 h	2134.2 (797.8)	2061.8 (1096.1)	0.95

Data are expressed as means (SD); *n* = 9 in each group and were analyzed by using the Wilcoxon signed-rank test

*iAUC* incremental area under the curve for test diet ingestion

The respiratory exchange ratio was slightly lower after ingestion of the modern Japanese diet than after ingestion of the traditional diet. Neither fat nor carbohydrate utilization during the experimental period (10 h) differed significantly between the test diets (data not shown).

**Fig. 3:**
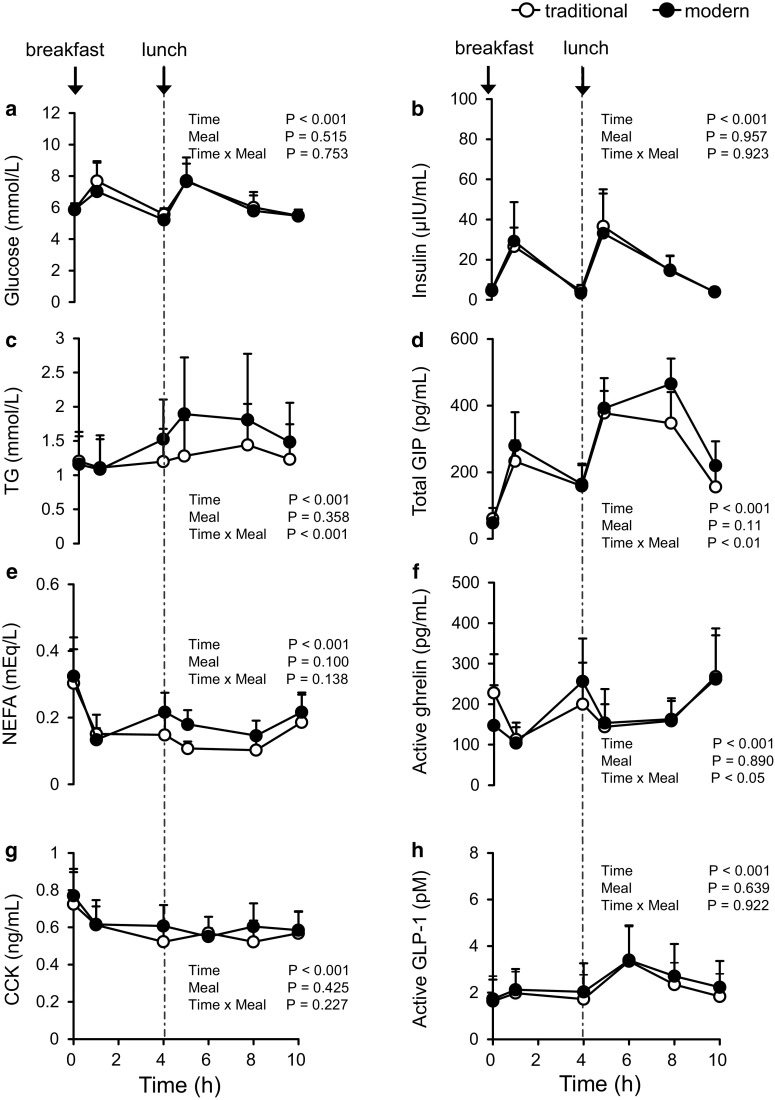
Blood glucose (**a**), insulin (**b**), triglycerides (TG; **c**), total glucose-dependent insulinotropic polypeptide (GIP; **d**), non-esterified fatty acids (NEFA; **e**), active ghrelin (**f**), cholecystokinin (CCK; **g**), and active glucagon-like peptide-1 (active GLP-1; **h**) concentrations until 10 h after the consumption of the traditional Japanese test diet (*open circles*) or modern Japanese test diet (*closed circles*) in overnight-fasted healthy Japanese men. Data are expressed as mean ± SD (*n* = 9 in each group) and were analyzed by means of 2-way repeated-measures ANOVA

As seen for the participants who ingested three meals, postprandial TG (*P* < 0.001) and total GIP (*P* < 0.01) differed significantly between diets among the second group of participants, who ingested two meals each (Fig. 3). Postprandial blood TG was significantly higher at 4 h after breakfast (*P* < 0.05) and at 1 h after lunch (*P* < 0.01) for the modern compared with the traditional Japanese diet. Postprandial blood GIP was significantly higher at 4 h (*P* < 0.01) and at 6 h (*P* < 0.05) after lunch for the modern compared with the traditional Japanese diet. The iAUCs for total GIP and active ghrelin after each meal (breakfast and lunch) (Table 3)—but not those for blood glucose, TG, NEFA, insulin, active GLP-1, and CCK concentrations (data not shown)—differed significantly (*P* < 0.05) between the test diets.

**Table 3:** Postprandial incremental areas under the curve (iAUCs) of total GIP and active ghrelin

		Traditional	Modern
*Total GIP*			
iAUC (after breakfast)	(ng 4 h/mL)	0.49 (0.14)	0.64 (0.18)
iAUC (after lunch)	(ng 6 h/mL)	0.91 (0.22)	1.27 (0.37)*
iAUC (after lunch): baseline 0 h	(ng 6 h/mL)	1.50 (0.31)	1.96 (0.24)*
*Active ghrelin*			
iAUC (after breakfast)	(ng 4 h/mL)	39.34 (82.30)	175.66 (128.94)*
iAUC (after lunch)	(ng 6 h/mL)	116.72 (200.85)	25.70 (50.75)
iAUC (after lunch): baseline 0 h	(ng 6 h/mL)	77.10 (94.96)	343.06 (236.90)*

Data are expressed as means (SD); *n* = 9 in each group and were analyzed by using the Wilcoxon signed-rank test

*GIP* glucose-dependent insulinotropic polypeptide

Values significantly (* *P* < 0.05) different from those of the traditional Japanese test diet are indicated

In addition to the effects on postprandial TG and GIP levels, the postprandial active ghrelin response showed a significant (Fig. 3f, *P* < 0.05) interaction between time and diet, even though the blood active ghrelin concentration did not differ between the test diets. There was no significant correlation between the postprandial GIP and active ghrelin responses.

### Association between postprandial REE and postprandial blood variables

**Fig. 4:**
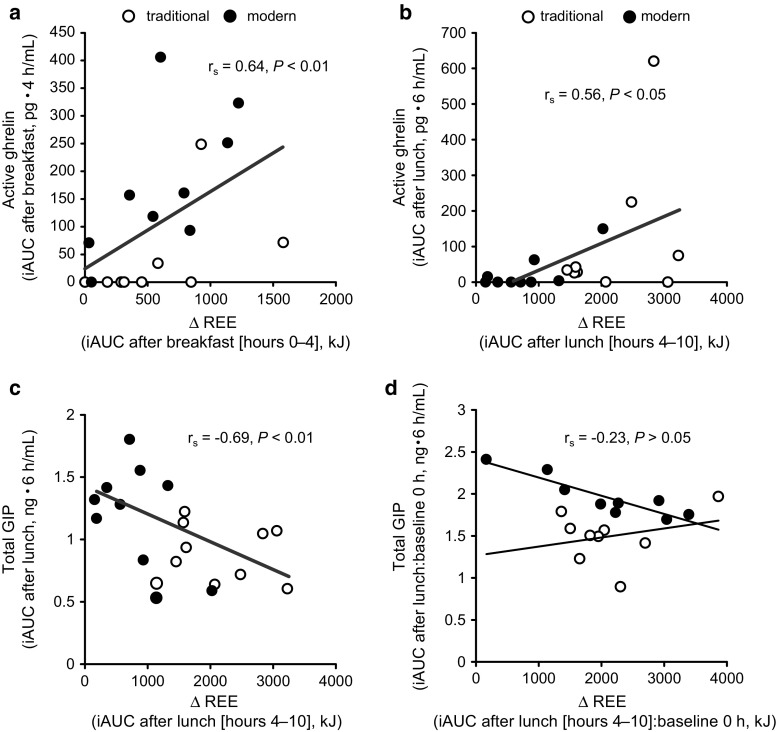
Correlation between the postprandial incremental area under the curve (iAUC) for ∆REE and postprandial blood levels of gastrointestinal hormones. **a** Correlation between iAUC-∆REE after breakfast and iAUC for active ghrelin. **b** Correlation between iAUC-∆REE after lunch and iAUC for active ghrelin. **c**, **d** Correlation of iAUC-∆REE after lunch with the iAUC for total GIP **c** when the values obtained immediately before ingestion of lunch (at 4 h) were used as baseline data and **d** when the values obtained immediately before breakfast (at 0 h) were used as the baseline levels. Spearman’s rank correlation coefficients were obtained when comparing paired samples whose data were not normally distributed. Correlations were considered significant when the error probability was <0.05

Correlation analysis revealed that the iAUCs for postprandial REE after breakfast and lunch were positively associated (breakfast: *r*_s_ = 0.64, *P* < 0.01; lunch: *r*_s_ = 0.56, *P* < 0.05) with the iAUC for postprandial active ghrelin (Table 4; Fig. 4a, b). The postprandial active ghrelin and REE responses were positively correlated with both the traditional (breakfast: *r*_s_ = 0.78, *P* < 0.05; lunch: *r*_s_ = 0.20, *P* > 0.05) and the modern (breakfast: *r*_s_ = 0.67, *P* = 0.06; lunch: *r*_s_ = 0.44, *P* > 0.05) diets. When the values immediately before breakfast (at 0 h) were used as the baseline levels, the iAUC of ∆REE after lunch was not positively correlated with the iAUC of active ghrelin after either the modern (*r*_s_ = 0.28, *P* > 0.05) or the traditional diet (*r*_s_ = 0.62, *P* > 0.05).

**Table 4:** Spearman’s rank correlation coefficients (*r*_s_) between postprandial iAUCs of REE and blood variable response

	Postprandial ∆REE (iAUC)	
	After breakfast 0–4 h	After lunch 4–10 h
*Total GIP*		
After breakfast 0–4 h	−0.07	–
After lunch 4–10 h: the levels at 4 h were used as baseline	–	−0.69^†^
After lunch 4–10 h: the levels at 0 h were used as baseline	–	−0.23
*Insulin*		
After breakfast 0–4 h	−0.23	–
After lunch 4–10 h: the levels at 4 h were used as baseline	–	0.05
After lunch 4–10 h: the levels at 0 h were used as baseline	–	−0.34
*Active GLP-1*		
After breakfast 0–4 h	0.27	–
After lunch 4–10 h: the levels at 4 h were used as baseline	–	−0.24
After lunch 4–10 h: the levels at 0 h were used as baseline	–	0.06
*Active ghrelin*		
After breakfast 0–4 h	0.64^†^	–
After lunch 4–10 h: the levels at 4 h were used as baseline	–	0.56*
After lunch 4–10 h: the levels at 0 h were used as baseline	–	0.31
*CCK*		
After breakfast 0–4 h	0.10	–
After lunch 4–10 h: the levels at 4 h were used as baseline	–	0.32
After lunch 4–10 h: the levels at 0 h were used as baseline	–	−0.01
*TG*		
After breakfast 0–4 h	0.03	–
After lunch 4–10 h: the levels at 4 h were used as baseline	–	−0.21
After lunch 4–10 h: the levels at 0 h were used as baseline	–	0.08

*CCK* cholecystokinin, *GIP* glucose-dependent insulinotropic polypeptide, *GLP-1* glucagon-like peptide-1, *TG* triglycerides, *iAUC* incremental area under the curve after the ingestion of test meals

Spearman’s rank correlation coefficients were obtained when comparing paired samples whose data were not normally distributed (* *P* < 0.05; † *P* < 0.01) between two parameters

The iAUC for postprandial REE after both lunches was negatively correlated (*r*_s_ = −0.69, *P* < 0.01) with the iAUC for the postprandial GIP response (Fig. 4c), when the values immediately before lunch (at 4 h) were used as the baseline levels (Table 4). When the values immediately before breakfast (at 0 h) were used as the baseline levels, the iAUC of ∆REE after lunch was negatively correlated with the iAUC of GIP after the modern Japanese diet (*r*_s_ = –0.83, *P* < 0.05) but not after the traditional diet (*r*_s_ = –0.15, *P* > 0.05) (Fig. 4d). There was no significant correlation between postprandial TG and REE responses (Table 4).

## Discussion

This study yielded two key findings. First, the study demonstrated that the nutritional composition of our modern Japanese test diet increased postprandial GIP, TG, and active ghrelin responses compared with those of the traditional diet in healthy Japanese male adults. Second, we showed that the modern Japanese meals tended to induce less postprandial energy expenditure than did the traditional meals, especially after lunch, and were associated with increased postprandial GIP release.

Postprandial GIP release is stimulated by macronutrients, especially dietary fat [27, 30–32]. The greater increase in postprandial GIP after the modern Japanese test diet seems to be due to its higher fat content compared with that of the traditional diet. In addition, the dietary fatty acid composition may have contributed to the increased GIP response after the modern meals. In the current study, the fatty acid composition (saturated/monounsaturated/polyunsaturated) was 5.9:6.7:9.1 g (27 %:31 %:42 %) for the traditional and 19.9:19.7:10.4 g (40 %:39 %:21 %) for the modern Japanese test diets. Monounsaturated fatty acids induce a stronger postprandial GIP response than do saturated fatty acids [30]. In addition, fish oil, which is rich in polyunsaturated fatty acids, suppresses the postprandial GIP response more effectively than do cocoa butter and olive oil [31]. Accordingly, the decreased polyunsaturated fatty acid content or the increased proportion of monounsaturated fatty acids (or both) may have contributed to the increase in the postprandial GIP response due to the modern Japanese test diet.

Like dietary fats, dietary protein increases postprandial GIP blood levels. Whey protein has been shown to stimulate postprandial GIP release into the blood [33]. Although the protein contents of the test diets were similar, the source of the protein differed between them: the traditional Japanese diet contained soy and fish, whereas the modern Japanese diet contained whey and meat as the main protein sources. The differences in the sources of dietary protein, which differed in amino acid composition, may have affected the postprandial GIP response. Additional studies are needed to clarify whether and how dietary protein differentially affects the postprandial GIP response in humans.

Regarding our second key finding, the results from our indirect calorimetry are consistent with the findings of Nagai et al. [34] and Maffeis et al. [35, 36], which showed that low-fat meals (energy %, 21, 20, and 27, respectively) induced greater thermogenesis postprandially than did extremely high-fat meals (energy %, 70, 48, and 52, respectively). Furthermore, our results parallel those from previous studies [34–36], even though the difference in the total fat content between the two test diets (energy %, 14.8 and 27.5) was relatively low in our study.

The postprandial energy expenditure due to dietary protein is 20–35 % of the energy consumption [37]. Several studies have suggested that high-protein diets lead to greater energy expenditure than do low-protein diets [38]. In the present study, the modern Japanese diet (13.1 % protein) tended to induce less energy expenditure than did the traditional diet (14.4 % protein), especially after lunch, even though the protein content of the diets was similar. The different sources of dietary protein (soy compared with milk) might be responsible for the difference between the postprandial energy expenditures of the test diets. Additional studies are needed to clarify whether and how dietary protein differentially affects postprandial thermogenesis in humans.

Accumulated evidence suggests that GIP, through its specific receptor, plays an important role in the onset of obesity by promoting energy storage in adipose tissues [18–26]. Several genome-wide association studies have identified single-nucleotide polymorphisms in the GIP receptor (GIPR) gene that are strongly associated with body mass index in humans [39–42]. Moreover, another recent study showed that the GIP/GIPR gene polymorphisms were strongly associated with visceral fat accumulation in the Japanese population [43]. In addition, increasing the blood concentration of GIP through its intravascular infusion decreased REE in healthy humans [21]. We previously showed that postprandial REE is negatively correlated with the postprandial GIP response and that a test diet that stimulated decreased postprandial GIP release was more thermogenic than was the control diet in healthy humans [44].

Like those involving human subjects, animal studies support the claim that decreased GIP signaling increases energy expenditure and suppresses body fat accumulation. Our previous study demonstrated that increasing blood GIP levels through chronic administration of GIP reduced fat utilization in high-fat fed mice [45]. The genetic ablation of either the GIPR [22] or of GIP-secreting enteroendocrine cells [20] prevented the onset of obesity and increased fat oxidation under high-fat diets through an unknown mechanism. The GIP receptor is expressed in pancreas, stomach, small intestine, heart, adrenal cortex, brain, lung, bone, vascular endothelium, and adipose tissue [46]. Although skeletal muscle and liver are the predominant sites of body fat oxidation, they do not express the GIP receptor. As a possible mechanism for linking GIP and energy catabolism, Naitoh et al. [47] showed that chronic inhibition of GIP signaling increased plasma adiponectin levels, resulting in increased fat oxidation in the skeletal muscle tissues of mice fed a high-fat diet. Decreased GIP secretion may increase energy expenditure through the stimulation of adiponectin signaling. However, whether this mechanism holds true during the postprandial state of humans is unclear, because the blood adiponectin level did not change after meal ingestion by human subjects [48]. Additional research is needed to clarify the role of adiponectin in linking postprandial GIP and energy expenditure in humans.

The GIP receptor is expressed in pituitary, adrenal cortex [46], and brown adipose tissue [49], all of which play important roles in energy homeostasis, including diet-induced thermogenesis. Therefore, as another possible mechanism linking postprandial GIP and energy expenditure, the increased GIP signaling after the modern Japanese diet may somehow modulate these tissues and thus downregulate energy expenditure. However, the physiologic function of GIP in pituitary, adrenal cortex, and brown adipose tissue in relation to postprandial energy expenditure is unclear and warrants investigation.

Blood concentrations of active ghrelin were positively correlated with the postprandial REE after breakfast and lunch, suggesting that, like GIP, postprandial ghrelin is a robust indicator of postprandial thermogenesis and the magnitude of the increase in active ghrelin seems to dictate the increase in postprandial energy expenditure. However, the postprandial increase in ghrelin was greater after the modern Japanese meal than the traditional meal—a finding that is incompatible with the result that the postprandial energy expenditure was lower after the modern Japanese diet than the traditional diet. Accordingly, the increase in the active ghrelin response does not seem to be predominantly responsible for the increased postprandial thermogenesis after the modern Japanese meals. Although increasing levels of active ghrelin during fasting has been shown to stimulate the appetite [13–16], the effect of ghrelin on energy expenditure has not been reported. Ghrelin increases hunger, food intake, and fat deposition by binding to its specific receptor in the ventromedial hypothalamus [50, 51]. In addition, electrical stimulation of the ventromedial hypothalamus of rats increased energy expenditure [52] and thermogenesis in brown adipose tissue [53]. Accordingly, ghrelin may increase energy expenditure through a hypothalamic mechanism. In one study [54], higher fasting blood ghrelin levels were associated with lower DIT, suggesting that ghrelin plays a significant role in regulating postprandial energy expenditure. In contrast, the role of the postprandial blood ghrelin response in regulating DIT remains to be clarified. In this regard, a high-protein diet was reported to increase postprandial thermogenesis but to blunt the postprandial blood ghrelin response [55]. However, blood ghrelin levels did not differ between the high-protein and the protein-sufficient diets in another experiment [56]. The relationship between the postprandial blood ghrelin response and DIT is therefore unresolved still.

The postprandial blood GIP response was negatively correlated with postprandial REE after the modern Japanese lunch but not after the traditional lunch (Fig. 4d). One possible explanation for this inverse correlation only after the modern Japanese lunch may be due to the concurrent increases in blood GIP and TG concentrations during and after the meal. Studies have shown that GIP increases the synthesis and secretion of lipoprotein lipase (LPL) in adipocytes [23, 57] and promotes the activity of LPL in fat tissue [58]. When the drop in LPL activity due to decreased GIP signaling in adipose tissue coincides with increased blood TG levels, the amount of fat released into the circulation may increase. In fact, ingestion of the modern Japanese diet (27.5 % fat content) increased the blood TG response more than twice that induced by the traditional diet (14.8 % fat content) (Fig. 1c), and the postprandial GIP response after the modern diet was almost double that after the traditional diet (Fig. 1d), supporting the hypothesis that concurrent increases in blood GIP and TG increase the amount of fat distributed to the circulation. When the postprandial GIP response is decreased in the context of increased blood TG, more fat may be distributed to energy-utilizing tissues such as skeletal muscle and liver, both of which lack the GIP receptor, to a greater extent than to energy-storing adipose tissues, thus increasing fat oxidation. However, the precise link between postprandial GIP signaling and whole-body energy utilization remains unclear and merits additional research.

Correlation analysis revealed that the iAUCs for the postprandial blood responses of CCK and active GLP-1 were not associated with postprandial REE in the current study, in contrast with the well-known correlation between GLP-1 and postprandial energy expenditure in other studies [59, 60]. The reason for these differing results is unclear but may reflect differences between the study protocols, including the dose of GLP-1. In addition, the postprandial increase in the blood level of active GLP-1 that we obtained in the current study was smaller than those in other studies [59, 60]. Additional work is needed to clarify the role of GLP-1 in postprandial energy expenditure.

According to the National Health and Nutrition Survey in Japan, the composition of the Japanese diet has changed markedly since 1960. However, the effect of dietary composition on energy homeostasis has been little investigated. The model composition of the average Japanese diet in 1975 was less obesity-inducing than was that in 2005 [61]. In the current study, the postprandial thermogenesis after the modern Japanese lunch was 1420 kJ less than that of the traditional Japanese lunch on average, which corresponds to 1.1 kg body fat per month. These results suggest that the nutritional change that accompanies the modernization of the Japanese dietary composition may increase body fat accumulation. The results of our current study also suggest that the postprandial GIP and active ghrelin responses are reliable predictors of postprandial REE and might be used as markers for designing obesity-preventive meals. In addition, our previous studies have consistently demonstrated that decreasing the diet-induced GIP response leads to increased energy expenditure and prevents high-fat diet-induced obesity in mice [44, 62].

A potential weakness of the present study is that our study population was small. Using a larger study population would be valuable for clarifying whether modern Japanese meals are less thermogenic than are traditional Japanese meals. Another limitation is that we did not address the mechanisms underlying the negative correlation between the postprandial GIP response and postprandial REE. Although GIP is well known as an incretin hormone that potentiates glucose-induced secretion of insulin from pancreatic β cells, blood insulin levels did not differ postprandially between the test diets. Accordingly, the extrapancreatic action of GIP—rather than its insulinotropic action—seems to facilitate the downregulation of postprandial energy expenditure. Additional studies are needed to clarify how blood GIP lowers postprandial thermogenesis. Another limitation of the present study is that the mechanisms underlying the positive correlation between the postprandial active ghrelin response and postprandial REE remain elusive. The contribution of the increase in ghrelin content to the decreased postprandial energy expenditure after the modern diet warrants further investigation.

In conclusion, the current study demonstrates that the modern Japanese test diet induced greater postprandial blood GIP and ghrelin levels than did traditional Japanese meals, and these hormonal responses were associated with postprandial thermogenesis in healthy Japanese men. We believe that not only the increased calorie intake but also the changes in the macronutrient composition that accompany the modernization of Japanese dietary patterns cause body weight and fat gain by altering the release of metabolism-regulating hormones to become more anabolic. The results of our study also indicate that the meal-induced release in the intestinal anabolic hormone GIP and the gastric hunger hormone ghrelin may be markers of postprandial thermogenesis and therefore may facilitate the design of obesity-preventative meals. The pairing of an increased postprandial ghrelin response with reduced the postprandial energy expenditure after the modern Japanese diet was inconsistent with the positive correlation between postprandial ghrelin response and energy expenditure; however, these data are inconclusive and merit further study. Our on-going studies seek to clarify whether and how the daily consumption of “low GIP meals” decreases body weight and fat mass in obese and overweight subjects. Additional detailed studies on the relationship among diet-induced GIP and ghrelin responses and postprandial energy homeostasis are warranted.

## Abbreviations

CCK: Cholecystokinin
GIP: Glucose-dependent insulinotropic polypeptide
GLP-1: Glucagon-like peptide-1
iAUC: Incremental area under the curve
NEFA: Non-esterified fatty acid
PP: Pancreatic polypeptide
PYY: Peptide YY
REE: Resting energy expenditure
TG: Triglycerides
VCO_2_: Carbon dioxide consumption
VO_2_: Oxygen consumption
